# Health Risk Assessment of Heavy Metals Accumulated on PM_2.5_ Fractioned Road Dust from Two Cities of Pakistan

**DOI:** 10.3390/ijerph17197124

**Published:** 2020-09-29

**Authors:** Haseeb Tufail Moryani, Shuqiong Kong, Jiangkun Du, Jianguo Bao

**Affiliations:** School of Environmental Studies, China University of Geosciences, Wuhan 430074, China; haseebmoriani@cug.edu.cn (H.T.M.); dujk@cug.edu.cn (J.D.)

**Keywords:** road dust, resuspension, toxic elements, inhalation, pollution assessment, cancer

## Abstract

The aim of this study is to identify and investigate levels of toxic heavy metals in PM_2.5_ fractioned road dust to better understand the associated inhalation risk and potential health impacts. To achieve this aim, concentrations of seven traffic generated heavy metals (Cu, Pb, Zn, Cd, Ni, Sb, and Cr) were determined in the PM_2.5_ fraction of road dust samples from four different locations (offices, residential, hospital, and school) in two cities (Karachi and Shikarpur) of Pakistan using ICP-MS. The average concentration values of heavy metals in Karachi were as follows: 332.9 mg/kg Cu, 426.6 mg/kg Pb, 4254.4 mg/kg Zn, 62.3 mg/kg Cd, 389.7 mg/kg Ni, 70.4 mg/kg Sb, 148.1 mg/kg Cr, whereas the average concentration values of heavy metals in Shikarpur were 245.8 mg/kg Cu, 538.4 mg/kg Pb, 8351.0 mg/kg Zn, 57.6 mg/kg Cd, 131.7 mg/kg Ni, 314.5 mg/kg Sb, 346.6 mg/kg Cr. The pollution level was assessed through two pollution indices enrichment factor (EF) and geoaccumulation index (I_geo_). These indices showed moderate to extreme level pollution in studied areas of both cities. The health risk assessment through inhalation contact was conducted according to the United States Environmental Protection Agency’s (USEPA) model for children and adults. Both non-cancerous and cancerous risks were characterised in the road dust samples for each location. As yet, there is not a single study on the concentrations of heavy metals in PM_2.5_ fractions of road dust in Karachi and Shikarpur, findings of this research will facilitate researchers for further investigations in current field.

## 1. Introduction

Road dust is known as a heterogeneous mixture of environmental pollutants originated from natural as well as anthropogenic sources. Among the natural sources, erosion, weathering, resuspension, and deposition of soil particles are considered the major road dust sources, whereas emissions from several activities of urbanisation such as traffic, industrial and household emissions are considered the main anthropogenic sources [1]. Due to large population sizes and presence of a variety of pollution sources, cities are more vulnerable to toxic road dust as compared to rural areas [2]. The association of detrimental health effects with pollutants present in the air of urban areas is a serious issue faced by the residents of urban areas [3]. In addition to a variety of other contaminants, road dust is a source of metal pollutants in urban areas, which can damage human health through ingestion, inhalation or dermal contact [4].

Resuspension of road dust is an important source of atmospheric particulate matter, particularly in the 2.5µm fraction (PM_2.5_) [5,6,7]. Resuspended road dust from paved and unpaved roads has been considered a significant contributor to PM_10_ and PM_2.5_ particularly in urban areas [8]. Reported by an earlier study road dust resuspension has been responsible for PM_10_, PM_2.5_, and PM_1_, respectively, for an average of 37 percent, 15 percent, and 3 percent of total traffic emissions [9]. Early studies suggested that particles of road dust with sizes below 100 μm can be readily resuspended by passing vehicles or wind [7]. Pollution from particulate matter has been considered a serious threat, especially for cities with heavy traffic [2], which is responsible for generating numerous metals like Fe, Cu, Zn, Sb, and Pb through exhaust and non-exhaust emissions [10]. Automobiles are the key contributors to the resuspension of road dust from heavy metals and other carcinogenic substances [11]. In addition, the presence of a large number of expired and poorly maintained automobiles in cities of developing countries also increases the level of PM_2.5_ pollution [12].

Heavy metal pollution is increasingly recognised as a serious, environmental issue by environmentalists [13]. High levels of toxicity, persistence, and potential for accumulation inside human body pose a serious health threat to the residents of urban areas [14,15]. A large variety of sources, such as vehicular, industrial, and domestic emissions play a significant role in increasing the concentration of heavy metals as well as several organic substances in urban environments [16,17]. Recent research has demonstrated that heavy metals in PM_2.5_, including (Pb, Cu, Ni, and Fe) collected from heavy traffic areas are capable of causing of several respiratory and cardiovascular diseases. Hence, even containing a slight portion of metals in PM_2.5_ can be injurious for human health [18].

In the last two decades, exposure to particulate matter through inhalation has become of considerable concern [19]. Research has shown PM_2.5_ pollution is most hazardous to the respiratory system as compared to other systems of the body [20]. Children are particularly sensitive to metal poisoning, which can negatively impact normal growth [16]. As the vitality of inhalation pathway is well established by previous research, the current study is focusing on the pollution of metals in the inhalable fraction PM_2.5_ of road dust. For this purpose, four areas were chosen including a hospital, offices buildings, school and residential area for road dust sampling from two cities, among them, Karachi is the largest city in Pakistan and second most populous city in South Asia, and Shikarpur is a small city of province Sindh in Pakistan. The motive behind choosing these areas was to focus on sites where a major portion of population interacts with road dust. Secondly, school and hospital areas were chosen in order to focus on places where children and patients, which have been considered as susceptible populations, may come in contact with road dust.

In Pakistan, a considerable amount of literature has been published on heavy metal pollution from road dust, including assessment of concentration levels [21], distribution patterns [22], their sources of origin [23], chemical composition and health impacts [24]. However, to date, no study that has assessed the presence of toxic metals in PM_2.5_ fractions from urban road dust samples in Karachi and Shikarpur. Road dust research as previously focused primarily on megacities, whereas very little attention has been given to road dust pollution in small cities [16,25,26]. Therefore, we chose Karachi and Shikarpur to compare the level of pollution between two cities that are different in size, infrastructure, and population. The growth of population and consumption of vehicles in Shikarpur with traffic infrastructure is relatively very low as compared to Karachi. In addition, there is inadequate mass transit in Shikapur, where narrow roads and an absence of sufficient traffic signals have led to a city overburdened with heavy traffic as compared to Karachi.

Therefore, the primary aims of this study are to (1) to determine the concentration level of seven common heavy metals (Cu, Pb, Zn, Cd, Ni, Sb, Cr) associated with traffic emissions from PM_2.5_ fractions of road dust in four different areas of Karachi and Shikarpur, (2) assess the level of pollution in each area by calculating pollution indices EF and I_geo_, and lastly (3) perform a health risk assessment for both children and adults according to hazard index (HI) and carcinogenic risk (CR) methods [27,28].

## 2. Materials and Methods

### 2.1. Study Area

Karachi and Shikarpur are two cities in the Sindh province of Pakistan. Karachi (24°51′ N 67° 02′ E) is located on the coast of Arabian Sea. The soil of Karachi is calcareous, marine in origin, and belongs to the upper Tertiary period [29]. It is the largest and most populous city in Pakistan and the 14th largest city in the world [30]. With a population of 24 million, it occupies an area of 3530 square kilometers with a vast road network of more than 8000 km [31]. The geology of Karachi consists of flat or rolling plains, surrounded by hills on northern and western part, with an undulating plain and extensive coastal area in the southeast. A vast area of about 300 km from north to south of Karachi is covered with hills of the Kirthar range, with the uppermost peak of almost 528 m on the northern side of the city [12]. The climate of the city is subtropical with an average rainfall of 256 mm and average monthly temperature ranging from 13 °C to 34 °C [32]. A recent study by K. Lurie (2019) has declared Karachi among cities with maximum health risk from total suspended particles (TSP) worldwide [12]. A total of 51% of the population of the city resides in slum areas, which are also main traffic centers of Karachi city [33]. Road dust and vehicular emissions are acknowledged as major agents responsible for heavy air pollution in the city of Karachi [34]. It has been estimated that Karachi has more than 3 million vehicles on the road per day [33]. The automobiles contributing to Karachi’s traffic are 30% motor bikes, 27% cars, 13% public transport, 12% trucks, 10% office vans, and 4% taxis and three- wheelers [35]. It has been also reported that the condition of 30% of vehicles in Karachi are not environmentally friendly. The sampling sites in Karachi were chosen from the major arteries where daily traffic volumes generally between 70,000 and 180,000 vehicles. In which motorbikes contributed a major portion of traffic followed by cars, buses, office vans, and three-wheelers.

**Figure 1:**
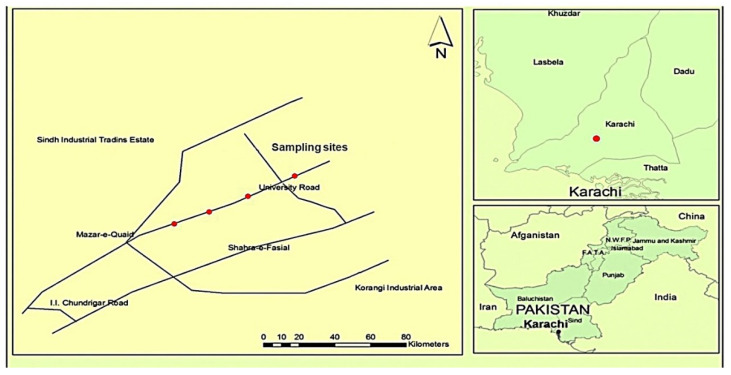
Sampling location map of Karachi (modified from [38]).

**Figure 2:**
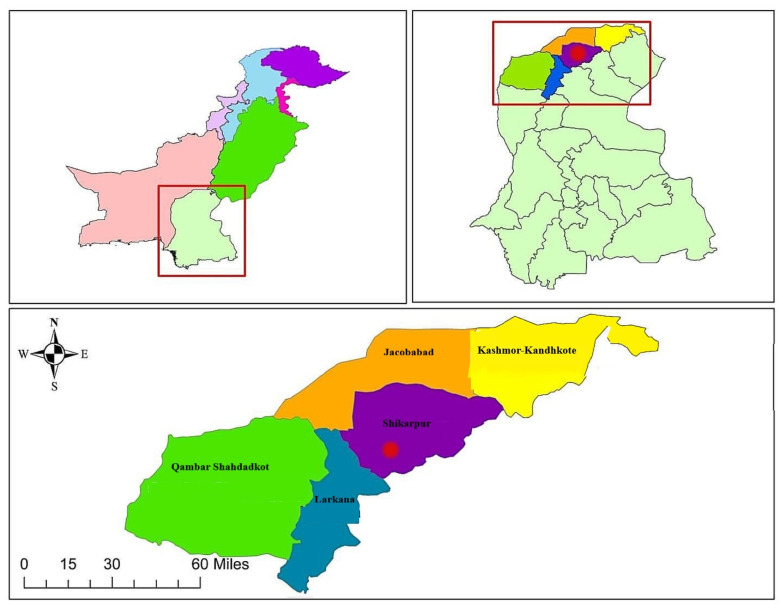
Sampling location map of Shikarpur.

In comparison, Shikarpur is a small agricultural city (27°57′25ʺ N 68° 38′ 16ʺ E) and the capital of the Shikarpur district is situated at the northern part of the Sindh province, approximately 29 km from the bank of the Indus river. The northern part of the Sindh province (which includes Shikarpur) is comprised primarily of sedimentary rocks of Laki formation and belongs to cretaceous and pre-cretaceous geological periods [36]. According to 2017 census, population of the Shikarpur district is 1,231,481, with 207,555 households. Among them, 303,249 people are living in urban areas, and the remainder are settled in rural areas [37]. Shikarpur plays a prominent role in rice production, due to this reason a large number of people from rural area are concerned with this work. There are no industrial activities in the vicinity of sampling area, and the largest sources of pollution are urban traffic on roads and in the residential area. The sampling sites in Shikarpur were chosen from the main road of Shikarpur, named as “station road” where daily traffic volumes generally between 7000 to 12,000 vehicles. In which motorbikes contributed a major portion of traffic followed by three-wheelers, cars, vans, and buses. Figure 1 and Figure 2 illustrates the sampling locations of this study.

### 2.2. Sample Collection

Four areas in each city were selected for road dust sampling including school, hospital, residential area and offices buildings. In each city sampling sites were selected from the areas with busy roads. The selection of these particular areas was done in order to cover maximum segments of our population included adults, children, and patients. Two locations from each site were selected for road dust collection. One sample was taken from the nearby roads and the second sample was taken in front of the main entrance of the area’s buildings, indicated as 1 and 2 in sample identity (ID), respectively. Classification of sampling locations on the basis of major activity (such as residential, offices, hospital and school) is displayed in Table 1. All study sites of present research were located on main roads in urban areas with heavy traffic. Road dust samples were collected from the hardened ground using a plastic brush and Pan, a commonly used method for collecting road dust samples in the literature [39,40]. Three replicate samples were taken from each sampling location, and then mixed up into one sample. The collected road dust samples were stored in plastic freezing bags. Amount of collected road dust sample from each site was >150 g. After road dust collection, all samples were taken to the laboratory and dried at room temperature. In order to separate dust from materials such as leaves, hair, cigarette butts, plastic particles etc., samples were sieved through 53 mesh and stored in air-tight plastic bags for analysis.

### 2.3. Preparation of PM_2.5_

In order to get the PM_2.5_ inhalable fraction of road dust 47 mm sized Teflon filters were used [41]. Before use, each 47 mm Teflon filter was dried at 150 °C using a vacuum freeze dryer to remove moisture for 6 h, conditioned for 48 h at 25 °C, and then blank filters were measured three times with electronic microbalance. The road dust particles <53 µm sized were poured in the 250 mL sized side-arm glass made flask fixed with a rubber stopper. After entering into the flask air pushed the dust into the resuspension chamber (Figure S1) and road dust samples were collected through PM_2.5_ inlets for about one minute on 47 mm Teflon filters [42]. In next step PM_2.5_ fraction contained filters were removed from inlet. After filtering the PM_2.5_ fraction, the weight of each filter was again measured three times with electronic microbalance. Finally, filters were folded in half, wrapped in aluminum foil sheets and stored at −20 °C until further analysis. Figure S2 shows the sampling scheme for the preparation of PM_2.5_ fractioned road dust.

### 2.4. Total Metal Concentration

Total metal concentration was determined using Inductively Coupled Plasma Mass Spectroscopy (ICP-MS, NexION-350X series) following aqua-regia digestion (MARS-6 microwave (CEM) and USEPA method 3051 (USEPA,1998)) with same procedure as a previous study [18]. Briefly, Teflon filters containing PM_2.5_ fraction of road dust samples were divided into two equal halves, each half was digested using 5ml of aqua regia solution. After digestion, process samples were diluted in a 2% nitric acid HNO_3_ solution for analysis with ICP-MS.

### 2.5. Quality Control

All samples were examined in triplicate including filter blanks and Standard reference material (SRM). Trace grade acids (hydrochloric and nitric acid) were used for digestion and analysis. The Standard reference material (GBW07451) was purchased from the National Center of Standard Materials of China (NCSMC). The analysis was performed in duplicate in order to confirm the accuracy of aqua-regia digestion method and spiked samples. The % recovery was calculated using concentrations of metals in SRM, spiked samples, and samples with same procedure as earlier studies [18]. Recoveries from internal standards and SRM ranged from (91.0% to 100.4%) and (80% to 130%), respectively.

### 2.6. Statistical Analyses

Statistical analyses were performed using Origin 9.0 (OriginLab Corporation, Roundhouse Plaza, Suite 303. Northampton, MA 01060, USA) and Microsoft excel 2016 (Microsoft Office, Las Vegas, NV, USA) softwares.

### 2.7. Pollution Assessment

Multiple methods have been applied to determine the levels of heavy metal pollution in natural environmental samples such as dust, soil, etc. In present study geoaccumulation index (I_geo_) and enrichment factor (EF) were employed to assess the level of heavy metal pollution in PM_2.5_ fractioned road dust samples. Index of geoaccumulation distinct from several other indices of pollution due to log function and constant factor of 1.5 to prevent lithogenic effects which may be related to fluctuations in baseline values [39]. While enrichment factor has been employed to differentiate the role of natural and artificial sources of pollution [43].

To compute the enrichment factor (EF) and geoaccumulation index (I_geo_) indices, background values of metals for Cu, Pb, Cr, and Zn, were taken from the baseline values of Karachi soils (Karim et al.) [44], which are 36.31 mg/kg, 56.23 mg/kg, 12.9 mg/kg, and 123.03 mg/kg, respectively. Whereas, background values for Cd and Ni were taken from the background values of Karachi soils from earlier study (Siddique et al.) [45], which are 7.42 mg/kg and 28.63 mg/kg, respectively. Due to the absence of Sb value in first two data sets, background value of Sb was used same as (Nekhoroshkov et al.) [46]. All background values were selected from those studies in which the uncontaminated soil samples for measuring baseline values were collected from 0 to 10 cm depth in order to avoid from surface contamination.

### 2.8. Geo-Accumulation Index (I_geo_)

In the 1960s, the Geoaccumulation Index (*I*_geo_) a most esteemed tool was introduced by (Muller et al.) [47] to quantify the level of heavy metal contamination in sediments. However, later on many researchers utilised this method for assessing the level of heavy metals contamination in soil and dust [21,48,49]. The *I*_geo_ is computed by the following equation:

(1) $$I_{\mathrm{geo}}=\;\log_{2}\frac{C_{\mathrm{HM}}}{1.5\;\times \mathrm{BV}}$$

where C_HM_ is the concentration of heavy metals in dust and BV is the background value of metal in soil. 

#### Enrichment Factor

Enrichment factor (EF) is an indicator which has been used to determine the intensity and availability of anthropogenic pollutants on the surface of soil and dust. We computed the enrichment factor to assess the enrichment values of heavy metals in road dust of Karachi and Shikarpur, following the equation reported by (Topi et al.) [50]. The contamination level was based on five categories of EF proposed by (Sutherland, 2000) [51].

(2) $$\mathrm{EF}=\frac{\mathrm{ACM}}{\mathrm{BVM}}$$

In the equation, ACM is average concentration of metal in particular area and BVM is background value of the same metal.

### 2.9. Health Risk Assessment

The accumulation of toxic metals in urban road dust may cause serious negative impacts on human health. The risk to human health posed by toxic metals can be quantitatively evaluated by recognizing the possible exposure outcomes [28]. Among the primary pathways of heavy metal exposure to human beings inhalation route has been given a prominent place [52]. In this study, health risks of PM_2.5_ fractioned road dust samples were assessed for children and adults by using two steps model i.e., exposure assessment and risk characterisation developed by (US EPA 1989; US EPA 2011) [16].

#### 2.9.1. Exposure Assessment

The present study focused on inhalation exposure only, and the exposure concentrations were calculated by following equation (Rehman et al.; Jayarathne et al.) [27,28].

(3) $$MDI_{inh}=\frac{C\times R_{inh}\times EF\times ED}{PEF\times BW\times AT}$$

In which *MDI_inh_* is the average metal daily intake dose for inhalation. *C* is concentration of metal in PM_2.5_ fractioned road dust samples. *R_inh_*, *EF*, and *ED* are rate of inhalation, exposure frequency, and exposure duration, respectively. *PEF* is particular emission rate. *BW* and *AT* are body weight and average time, respectively. 

#### 2.9.2. Risk Assessment

Health risk assessment for Children and adults was performed by calculating hazards quotient (*HQ*), hazards index (*HI*), and carcinogenic risk index (CRI). Among these *HQ* and HI describe non-carcinogenic risk, whereas CRI shows the probability of the potential of heavy metals for cancer risk to children and adults. The Equations (4) to (6) were used to calculate these three parameters.

(4) $$HQ=\frac{MDI\;}{RFD}$$

(5) $$HI=\sum^{\text{​}}HQ_{i}$$

(6) CR=MDI ×SF

In Equation (4) *MDI* and *RFD* are metal daily intake dose and reference dose, respectively. In Equation (5) *HQ* denotes non-carcinogenic risk and *HI* is the sum of hazards quotient of inhalation. In Equation (6) *CR* explain carcinogenic risk whereas, *MDI* and *SF* indicate metal daily intake dose and slope factor, respectively. The values of reference dose (*RFD*) for inhalation exposure pathway and slope factors are listed in Table S1. The health risk assessment result for a child and an adult for inhalation route at each studied area are arranged in Tables S2 and S3.

## 3. Results and Discussion

### 3.1. Heavy Metal Concentration in Road Dust

**Figure 3:**
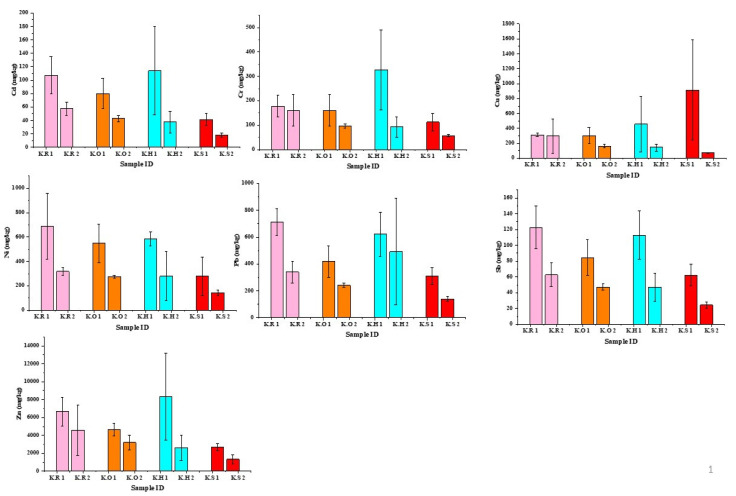
Concentration of metals (mg/kg) in PM_2.5_ fraction of road dust from four areas of Karachi (K.R: Karachi residential area; K.O: Karachi offices area; K.H: Karachi hospital area; K.S: Karachi school area). Data represent the mean ± STD.

**Figure 4:**
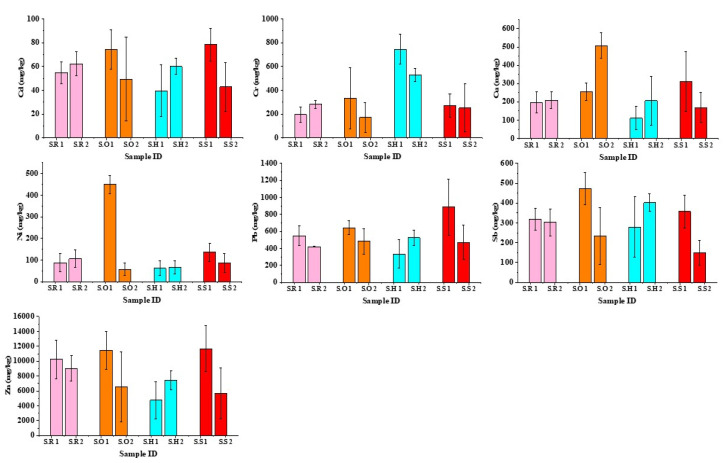
Concentration of metals (mg/kg) in PM_2.5_ fraction of road dust from four areas of Shikarpur (S.R: Shikarpur residential area; S.O: Shikarpur offices area; S.H: Shikarpur hospital area; S.S: Shikapur school area). Data represent the mean ± STD.

Concentrations of Cu, Pb, Zn, Cd, Ni, Sb, Cr were measured in PM_2.5_ fraction of road dust samples from four areas (hospital, school, offices, and residential) of Karachi and Shikarpur, Pakistan. The minimum, maximum, mean and standard deviation (SD) of total metal concentrations associated with PM_2.5_ fraction are summarized in Table 2 and Table 3 and compared to other major Asian cities in Table 4. The variations in the concentration of metals between the locations of both cities are shown in Figure 3 and Figure 4. The highest mean concentration values in the samples of Karachi were found for Zn followed by Pb, Ni, Cu, Cr, Sb, and Cd, respectively, whereas in the samples of Shikarpur, mean concentration of heavy metals in descending order was found as Zn, Pb, Cu, Cr, Sb, Ni, and Cd. However, on the basis of land use types the descending order with respect to average concentration values of toxic metals in Karachi and Shikarpur ranked as residential area > hospital area > offices area > school area and residential area > offices area > school area > hospital area, respectively. These results seem to be consistent with the observations of other researchers, which also found a significant amount of Zn, Pb, Cu, and other elements in road dust [42,53].

Pb exposure can affect human health in various ways such as neurological disorders in children, malfunction of kidneys, cardiovascular problems, genotoxicity, and disruption of stress mechanisms which can cause neurotoxicity [56,57]. Incidental ingestion and inhalation of soil/dust particles are considered major sources of Pb exposure in human beings [57]. The heavy concentration of Pb in road dust samples of both cities indicates traffic as a major source of pollution. The concentration of Pb for each area in Karachi followed the order residential area > hospital area > offices area > school area, whereas for Shikarpur the concentrations were school area > offices area > residential area > hospital area. The differences in the concentration of Pb in PM_2.5_ fractioned road dust in all areas can be related to the volume of vehicles. Data presented in Table 5 shows fuel combustion, tyre, and brake wear emissions, as major contributing factors for Pb in road dust. Since the termination of leaded petrol phase, tyre loss emissions has been identified as a large contributor of Pb in road dust [58]. Despite restrictions on use of leaded gasoline, it continues to be an environmental health risk for at-risk populations (e.g., children) [59]. Since 2005 leaded petrol has been banned in Pakistan, but illegal intrusion of leaded petrol in Pakistan from neighboring countries has been regarded as one of the major cause of high concentration of Pb in road dust [23]. The excessive use of leaded gasoline in addition to poor sanitation systems have played an active role in high concentration levels of Pb in road dust of Shikarpur. To date, only few studies have been attempted to investigate heavy metal concentration in PM_2.5_ fractioned road dust, which are set out in Table 4. It can be seen that the concentration of Pb measured in the road dust of Karachi is higher in two cities out of four, whereas one out of four has higher concentration than that of Shikarpur. Comparison of the concentration of Pb in road dust samples from urban traffic zones in Hong Kong recorded by (Ho et al.) [55], where higher average concentration of Pb was found as compared to this study with the indication that traffic exhaust has a significant contribution in the high concentrations of Pb in urban road dust.

Humans are exposed to copper particles through four major pathways: inhalation, ocular absorption, dermal absorption, and ingestion [65]. Among these four pathways, inhalation is of most concern both because it is difficult to avoid and because the very small size allows deep penetration into lung tissues, resulting in several health issues like oxidative stress, inflammation, and accumulation in various organs through blood transportation [65,66]. The concentration of Cu for the areas of Karachi in decreasing order was school area > hospital area > residential area > offices area, whereas for Shikarpur offices area > school area > residential area > hospital area. The highest concentrations of Cu were measured in the samples of the school area of Karachi (913.3 mg/kg) and offices area of Shikarpur (507.6 mg/kg). High concentration of Cu in the road dust of school and offices areas of both cities can be related to the high frequency of braking due to the sensitivity of the area. Previous studies have characterised Cu in road dust as traffic-generated and have identified non-exhaust emissions especially from tyre and brake wear as a major source (Table 5). In Europe, it has been recorded that about 2400 tons of copper were released from brake wear in 2000, accounting for 48% of copper emitted by all sources [61]. As the Cu is a major ingredient of brake pads used as a friction material in order to make smooth braking, high concentrations of Cu in heavy traffic areas of this study is consistent with the active role of brake wear emissions. Moreover, overuse of brakes due to heavy traffic can be understood as a contributor to the variation in the concentration of Cu in road dust. Karachi is the largest industrial city of Pakistan, therefore beside traffic emissions active role of industrial emissions cannot be denied in case of Karachi. Data documented from previous literature in Table 4 shows that two out four cities have lower concentration of Cu in PM_2.5_ fractioned road dust than that of Karachi and Shikarpur.

The highest average concentration of Zn in the samples of Karachi and Shikarpur was 4254.4 and 8351.0, respectively. In terms of studied areas, concentration of Zn for Karachi in decreasing trend was hospital area > residential area > offices area > school area, whereas for Shikarpur school area > offices area > residential area > hospital area. Of the seven metals assessed in this study, Zn was highest in concentration in all study areas of both cities and indicates that traffic is the only source of pollution in these areas. The tyre sector represents the largest single market for zinc oxide, producing over half of the world’s total demand of 1.2 million tons [61]. It has been reported in earlier study that a tyre discharges almost 1.5 kg of wear emissions in environment during its life span [67]. Previous researchers have found brake and tyre abrasion, greasing oil burns and wastes, as the dominant contributors of Zn in urban road dust (Table 5). The high frequency of braking is a common practice in the urban cities laden with heavy traffic, which consequently leads to the generation of high amounts of Zn in road dust samples in Karachi and Shikarpur. These results seem to be consistent with previous literature given in Table 4, which also shows the highest concentration of Zn among all seven metals.

The concentration of Cd in descending order for the studied areas of Karachi and Shikarpur was same as Zn. Previous study has indicated a significant role of several agents such as wear from brakes, tyres, engine wear, oil leakage and auto printing in the high concentration of Cd in road dust (Table 5). Beside this, there can be several reasons behind the high concentration of Cd in road dust samples from Karachi and Shikarpur, such as heavy traffic flow, waste from batteries and alloys, diesel exhaust, poor maintenance of vehicles, unstable condition of roads etc., which play a dominant role in generating higher amount of heavy metals in road dust. Consistent with the previous literature documented in Table 4, results of current study also showed the lowest concentration of Cd among all seven metals, except Hong Kong, where a significant amount of Cd has been found in PM_2.5_ fractioned road dust. In comparison to other major cities (Table 4), Karachi and Shikarpur had the second highest concentration of Cd in PM_2.5_ fraction of road dust following Hong Kong.

In terms of studied areas, concentration of Ni for Karachi followed the sequence residential area > hospital area > offices area > school area, whereas for Shikarpur offices area > school area > residential area > hospital area. Data documented in Table 5 demonstrates the association of Ni in road dust with fuel combustion and tyre abrasion. Variation in the concentration of Ni at different sites can be related to the burden of traffic as tailpipe emissions and fuel combustion are considered major sources of Ni in road dust. Additionally, old and poorly maintained vehicles also contribute to Ni concentrations in road dust through decomposition of their parts and fuel combustion, as well as emissions from tyre abrasion. Another prominent source of higher Ni concentration in Karachi can be the intense ship traffic, as ship emissions has been considered as a significant source of Ni [68]. Table 4 shows the concentration of Ni in PM_2.5_ fractioned road dust for 4 major cities of the Asia. Of these 4 cities, Karachi was highest for Ni concentration, and Shikarpur lies on the second position.

Sb is toxic to humans and has been characterised as a carcinogen. Although the concentrations of Sb in road dusts are much lower than Cu and Zn, the high toxicity of Sb has made it a primary focus by researchers. The concentration values of Sb for the areas of Karachi followed the sequence residential area > hospital area > offices area > school area, whereas for Shikarpur offices area > hospital area > school area > residential area. Sb is a major component of road dust, and its presence in road dust has been connected with the exhaust as well as non-exhaust sources of traffic emissions such as tailpipe emission, brake wear, and tyre wear (Table 5). High levels of Sb in the samples from the main road of the residential area may be related to the action of repeatedly applying brakes, due to the sensitivity of area. Data presented in Table 4 shows that the Sb was found in PM_2.5_ fractioned road dust of two out of four cities, and all values were lower than Shikarpur but higher than that of Karachi. As a small city, Shikarpur is more congested with traffic as compared to Karachi therefore, the high concentration of Sb in Shikarpur can be related to factors such as high frequency of braking, poor condition of tyre, and usage of low-quality fuel.

Based on the studied areas concentration values of Cr for Karachi showed the sequence was hospital area > residential area > offices area > school area, whereas for Shikarpur hospital area > offices area > residential area > school area. The highest concentrations of Cr 326.3 mg/kg and 744 mg/kg were found in the samples from the hospital areas of Karachi and Shikarpur. The lowest concentration of Cr (57.6 mg/kg) was found in samples from the school area of Karachi. Considering the differences in the concentrations of Cr at these different locations active role of two-stroke engine vehicles cannot deny which is biggest portion of Karachi’s traffic almost cover 30% of whole [35]. Previous research has shown that two-stroke engines emit 13 times more exhaust as compared to four-stroke engines [69]. In addition to this, previous study has been reported fuel combustion and brake wear as a significant source of Cr (Table 5). Comparison of the findings with the data reported in Table 4 shows that Shikarpur has second highest average concentration of Cr in road dust following Incheon (Korea), whereas Karachi ranked on third position following Incheon and Hong Kong. High concentrations of Cr in urban road dust of Thessaloniki have been linked to material produced as the result of brake friction, and is considered to pose a potential risk to the health of children [70].

### 3.2. Assessment of Pollution Level

Several techniques have been suggested for quantifying the level of metal contamination in dust and soil. We used the enrichment factor (EF) and geoaccumulation index (I_geo_) indices to assess the level of heavy metal contamination in the PM_2.5_ fraction of road dust collected from four areas of Karachi and Shikarpur. EF and I_geo_ indices have been considered as well established methodologies for quantifying the effects of heavy metals on environment by previous research [50,71]. The categories of geoaccumulation index and enrichment factor are classified in Tables S4 and S5, respectively.

#### 3.2.1. Enrichment Factor (EF)

The computed enrichment factor results for Karachi and Shikarpur are shown in Table 6 and Table 7, respectively. The EF ranges of Cu, Pb, Zn, Cd, Ni, Sb, Cr for Karachi and Shikarpur were 1.9–25.1, 2.5–12.6, 10.8–67.4, 2.4–15.3, 4–24, 40.7–204.2, 4.4–25.3 and 3–13.9, 5.8–15.7, 38.4–94.9, 5.3–10.5, 2–15.6, 376.3–1183.1, 13.2–57.6, respectively. As per given criteria listed in Table S5 the enrichment level of studied heavy metals in the PM_2.5_ fractioned road dust of Karachi and Shikarpur was from minimal to extreme level. The maximum EF values of Zn and Sb in Karachi and Zn, Sb, Cr in Shikarpur were higher than 40, which illustrated the relation of metals with anthropogenic sources. The high accumulations of Cu, Pb, Zn, Cd, Ni, Sb, and Cr in the road dust of studied areas of both cities showed their origin from traffic emissions [72]. The pollution level of Sb in the studied areas of both cities was found significantly higher than other metals, this result connects the elevated concentration of Sb with artificial sources. This higher concentration of Sb in urban road dust can be relate with the several exhaust and non-exhaust traffic emission sources such as tailpipe, brake wear, and tyre wear. The highest calculated EF value of Zn (94.96) from Shikarpur is comparable with the previous study by (joshi et al.) [73] where it was recorded as (>100) and designated its origin from anthropogenic sources. An EF value >10 from all four studied areas of both cities for each studied metal is a clear indication that traffic emissions are a major source of enrichment for these toxic metals in road dust.

#### 3.2.2. Geoaccumulation Index (*I*_geo_)

The Geoaccumulation Index (*I*_geo_) was calculated for all seven metals and minimum, maximum, and average of the calculated values can be seen in Table 8 and Table 9. The average *I*_geo_ values of Pb have shown moderately polluted environment in the road dust samples of studied areas of Karachi. Whereas, moderately to strongly polluted environment in the samples of studied areas of Shikarpur. The average *I*_geo_ result for Cu shows moderately to strongly polluted environments in road dust samples of all four research locations of Karachi and Shikarpur. Likewise, Cu in the case of Cd the mean *I*_geo_ values have shown moderately to strongly polluted environment in the samples of studied areas of Karachi as well as Shikarpur. The mean *I*_geo_ values for Ni are indicating strong pollution in the samples of studied areas of current research in Karachi. Whereas, moderate pollution in the samples from Shikarpur. The average *I*_geo_ for Cr shows moderate to strongly polluted environment in the studied areas of Karachi. Whereas, strong to extremely polluted environment for the researched areas of Shikarpur. In the case of Zn road dust samples shows strongly to extremely polluted and extremely polluted for the studied areas of Karachi and Shikarpur, respectively. *I*_geo_ values for Sb have indicated extreme pollution in all four areas of both cities studied in this research. On the basis of *I*_geo_ results, it can be seen that the environment of particular areas studied in this research are highly polluted by metals besides this extremely high enrichment was noted for Sb and Zn.

### 3.3. Health Risk Assessment of Resuspended Road Dust

Road dust laden with toxic metals is capable of posing health risks to susceptible populations with weak immune systems such as children and patients. Health risk assessments of the studied heavy metals in the PM_2.5_ fractioned road dust samples of Karachi and Shikarpur through inhalation exposure was performed for children and adults. The non-carcinogenic and carcinogenic health risks related to studied metals for children and adults were computed by employing USEPA health risk assessment method. As the local guideline values are unavailable, so in order to calculate health risks using USEPA’s model values were taken from previous literature and documented in Table 10. The hazard quotient (HQ) and hazard index (HI) of non-carcinogenic and carcinogenic health risks were estimated by employing MDI_inh_ values of toxic metals exposed to children and adults via resuspended road dust. In the present study six toxic metals (Pb, Cu, Cd, Ni, Cr, and Zn) were selected for non-carcinogenic and five metals (Pb, Cd, Ni, Cr, and Sb) were selected for carcinogenic health risk assessment. This study revealed higher health risks to adults through inhalation route than children, which can be related to greater exposure duration and inhalation rate of adults than children.

#### 3.3.1. Non-Carcinogenic Risk

The present study revealed that the HI values of non-carcinogenic health risk for analysed metals for children in the studied areas of Karachi and Shikarpur were in the order of Cu > Cr > Pb > Cd > Ni > Zn and Cu > Cr > Pb > Cd > Zn > Ni, respectively. Whereas, the HI (non-carcinogenic) values for adults in the studied areas of Karachi and Shikarpur were in the order of Ni > Zn > Cu > Cr > Pb > Cd and Ni > Cu > Cr > Pb > Cd > Zn, respectively.

Table S2 displays the health risk results of non-carcinogenic and carcinogenic by HQ, whereas HI, and CR (cancer risk) values are presented in Table S3. The data of present study has revealed that HI (non-carcinogenic) values of all studied metals from Karachi and Shikarpur for children were found <1 except Cu (8.25 × 10^−6^) whose values exceeded from the safe limit (1) established by (USEPA 1989) [16]. Whereas, in the case of adults HI (non-carcinogenic) values of Cu (3.42 × 10^−6^), Ni (7.82 × 10^−6^), Zn (5.86 × 10^−6^) from Karachi and Cu (2.53 × 10^−6^), Ni (2.64 × 10^−6^) from Shikarpur were found exceeded from safe limit. Considering the findings of this study it has been suggested that if children are contacted by the PM_2.5_ fractioned road dust of Karachi and Shikarpur in a heavy amount through inhalation pathway then it can become the solid cause of lung infections due to copper contamination. It is necessary here to note that heavy amount of Ni, Zn, and Cu in inhalable fraction of road dust in urban areas is capable to affect human health in multiple ways for instant affecting respiratory system, kidneys, bones and DNA function. These results therefore suggested that Ni, Zn, and Cu in inhalable fraction of road dust from Karachi and Shikarpur should not be ignored.

Furthermore, HI (Children) values for Pb, Cd, Ni, Cr, and Zn in road dust samples of Karachi and Shikarpur are less than 1, indicating low health risks from road dust in studied areas. As heavy metals are capable to accumulate in the body and can stay for a long time span, in such case the heavy metals like Cd, Ni, Cr might be the because of several non-carcinogenic effects for adults.

#### 3.3.2. Carcinogenic Risk

The probability of developing any sort of cancer to a person from lifetime exposure to carcinogenic hazards is defined as carcinogenic risk [16]. In the present study cancer risk for Pb, Cd, Ni, Cr, and Sb was computed by multiplying average metal daily intake dose for inhalation (MDI_inh_) with slope factor and results are displayed in Tables S2 and S3. The highest HI (CR) values for children and adults in Karachi were recorded in the order of Cd > Pb > Ni > Cr > Sb and Cr > Cd > Pb > Ni > Sb, respectively. Whereas, the HI (CR) values for children and adults in Shikarpur were found in the decreasing trend of Pb > Cd > Ni > Cr > Sb and Cr > Ni > Pb > Cd > Sb, respectively. The reason behind a higher probability of cancer risks to adults through than inhalation route than to children was due to their greater exposure duration and inhalation rate. Cr has been considered as a most prominent element among all the metals selected for computing carcinogenic health risk in present study due to its carcinogenic toxicity. The computed HI (CR) values of Cr and Sb for children at the sampling sites of Karachi and Shikarpur were found <1, which shows negligible cancerous health risks to Children in studied areas of both cities. HI (CR) values of Cr for adults (2.57 × 10^−6^) and (6.02 × 10^−6^) in Karachi and Shikarpur, respectively were found exceeded from the safe limits, which indicates major cancerous health risks to adults in the selected research areas. Whereas, HI (CR) values of Sb for adults showed negligible cancerous risk at the sampling sites of both cities. Beside Cr the HI (CR) values of Pb, Cd and Ni for children and adults in the selected areas of both cities were found exceeded from the safe limit, these results clearly indicate higher cancer risk by these metals through inhalation pathway in studied areas. These findings are also consistent with the results of pollution index (I_geo_), which shows moderate to strong pollution of Pb, Cd and Ni in studied areas of current research. Therefore, it has been assumed that the concentrations of Pb, Cd and Ni in selected areas of Karachi and Shikarpur are considered as a cancer risk. While the HI (CR) values of Cr were estimated within safe limits for children, but very high carcinogenic risk was indicated for adults in the studied areas of both cities.

### 3.4. Possible Factors Responsible for High Concentrations of Metals in Road Dust in Karachi and Shikarpur

As shown in Table 2 and Table 3, the average concentrations of seven metals studied in this research are much higher than some cities reported in the literature (Table 4). These high concentrations of toxic metals in the road dust of Karachi and Shikarpur can correlate with multiple reasons. Karachi is the most urbanised and industrialised city of Pakistan, with largest seaport and airport. Due to these reasons a considerable number of people from all over the country are coming to Karachi for earning their bread, which is a big reason for rapid increase in the population. As the number of people is increasing in the city, simultaneously the number of vehicles is also increasing. According to the (Imran Ayub, DAWN 2017: https://www.dawn.com/news/1370976) everyday, 700 new bikes are becoming part of Karachi’s traffic, which plays a significant role in deteriorating road dust. Another eminent cause of road dust contamination is huge number of poorly maintained automobiles. Majority drivers of three-wheeled vehicles (M3W) in Karachi are not the owners of those vehicles, due to this they do not give proper attention towards the maintenance. Consequently, malpractices like adulteration in fuel and using low-quality fuel is a common practice to them. It has been reported by earlier studies that 30% of vehicles on the roads of Karachi does not meet the required criteria for sustainable environment [23]. Reported by (Zafar et al.) [77] two-third portion of Karachi’s traffic comprised of two-wheeled vehicles, especially two-stroke engine vehicles, which are more unfriendly towards environment as compare to four stroke engines. In addition to this, availability, and use of low-quality fuel illegally imported from the neighboring countries, is also a leading cause of high concentration of toxic elements in road dust of Karachi and Shikarpur. However, the sampling of the present study in both cities was done from main roads with heavy traffic away from the industrial zones, but the role of industrial emissions and waste cannot be totally avoided specially in case of Karachi which is the largest industrial hub of Pakistan. Here it can be notice that the concentration values of Pb, Zn, Sb, Cr in the road dust samples of Shikarpur were found higher than that of Karachi, these higher values can be relate with several factors for instance road erosions, poor urban planning, absence of an adequate garbage and waste disposal system which have turned Shikapur into the Pakistan’s filthiest city. As a number of studies have considered the effects of resuspended road dust injurious to human health, this first study on resuspended road dust of Karachi and Shikarpur will encourage the researchers to carry out further research to explore the risks of heavy metals concerned with inhalable fractions of road dust.

### 3.5. Comparison of the Health Risk Assessment Results with Other Cities

The toxic heavy metals associated with the inhalable fraction of dust has been considered as serious hazard towards human health [19]. In our study, no significant non-carcinogenic health risk to children were found due to Cd, Pb, and Zn which is similar to the findings of studies on Lahore [27] and Nanjing [78]. The non-carcinogenic health risk values due to exposure of Ni and Cr to children were found within the safe limit, which is consistent with the results of previous study on Beijing [4]. The present study shows no significant non-carcinogenic health risk to adults in the areas of Karachi and Shikarpur due to all metals except Cu and Ni, this finding is consistent with that of Hefei [1] where HQ_inh_ values of Cu and Ni were found higher than other metals. In accordance with the present results, previous study on Beijing [2] has also found high CR values of Ni for adults and children and Pb for adults than the acceptable limit. This study have found CR values of Cr for children within the tolerable range, these results are in agreement with those obtained by previous study on Guiyu, China [79]. Similar with the findings of previous study [80], results of this study also show higher carcinogenic risks due to exposure of Cd, Ni, and Pb to both children and adults. For some metals our study revealed values of HIs and CRs of inhalation higher for adults than children, this finding is consistent with the recent study on Sistan, Iran [81]. The cancer risk for an inhalation exposure of Sb was found negligible to children and adults in all sampling sites, this result is in accord with recent study on Bogota [82]. It is worth noting that although all mentioned cities are major cities, but due to difference of sampling areas and geochemical composition of dust, the results may not accurately demonstrate the difference between cities. However, there is a rough understanding of the similarities between them.

## 4. Conclusions

Road dust is a serious issue for residents of urban areas due to its acute toxicity and easy exposure to humans through inhalation, ingestion, and dermal contact, which consequently can cause several diseases. The present study aimed to determine the concentrations of seven metals (Cu, Pb, Zn, Cd, Ni, Sb, Cr) in PM_2.5_ fraction of road dust from four different locations (hospital area, offices area, school area and residential area) of Karachi and Shikarpur. The contamination levels of many metals calculated in present study are higher than those other megacities. Heavy traffic, poor maintenance of vehicles, and huge number of two-stroke engine vehicles are a major source of the high concentration of metals in the road dust of Karachi and Shikarpur. Specifically, high concentrations of Cr, a known carcinogen in hospital area, as well as Cu in the school area, indicate hazardous metal pollution. Assessment of the pollution level of metals in the road dust samples using enrichment factor (EF) and geoaccumulation index (*I*_geo_) indices showed moderate to extreme level of pollution in all four areas of both cities. The HI values indicated an adverse non-cancerous health risk for children due to Cu contamination and a significant non-cancerous health risk for adults to Cu, Ni, Zn in Karachi and Cu, Ni in the study areas of Shikarpur. For carcinogenic health risk, the HI values exceeded the tolerable range for Pb, Cd, and Ni, indicating heavy cancer risk for children in the study areas of both cities. Moreover, in addition to Pb, Cd, and Ni, the estimated HI values of Cr for adults were found to be higher than the limited margin, which indicates heavy cancer risk for adults due to road dust exposure through inhalation in all studied areas. This report calls for protective policy changes to directly address the heavy metal pollution of road dust in Karachi and Shikarpur. It is highly recommended that particular attention should be given to traffic pollution of areas containing schools and hospitals in order to protect at-risk populations from the harmful effects of heavy metal pollution. These data provide a framework for reducing metal pollution in road dust in studied areas and other megacities worldwide.

## Figures and Tables

**Table 1:** Sampling locations and characteristics.

Sampling City	Abbreviation	Nature of Road	Activity
Karachi	K.R1	Major road	Residential
Karachi	K.R2	Gate of residential building along the major road	Residential
Karachi	K.O1	Major road	Offices
Karachi	K.O2	Gate of offices building along the major road	Offices
Karachi	K.H1	Major road	Hospital
Karachi	K.H2	Gate of hospital along the major road	Hospital
Karachi	K.S1	Major road	School
Karachi	K.S2	Gate of school along the major road	School
Shikarpur	S.R1	Major road	Residential
Shikarpur	S.R2	Gate of residential building along the major road	Residential
Shikarpur	S.O1	Major road	Offices
Shikarpur	S.O2	Gate of offices building along the major road	Offices
Shikarpur	S.H1	Major road	Hospital
Shikarpur	S.H2	Gate of hospital along the major road	Hospital
Shikarpur	S.S1	Major road	School
Shikarpur	S.S2	Gate of school along the major road	School

**Table 2:** Heavy metal concentration (mg/kg) in PM_2.5_ fraction of road dust of all four areas of Karachi.

Statistical Values	Concentration (mg/kg)						
	Cu	Pb	Zn	Cd	Ni	Sb	Cr
Minimum	70.9	140.5	1332.3	17.9	142.6	24.4	57.6
Maximum	913.3	712.3	8299	114.1	689	122.5	326.3
Mean	332.9	426.6	4254.4	62.3	389.7	70.4	148.1
S.D	347.4	220.7	2853.5	40.4	219.2	36	98.8

**Table 3:** Heavy metal concentration (mg/kg) in PM_2.5_ fraction of road dust of all four areas of Shikarpur.

Statistical Values	Concentration (mg/kg)						
	Cu	Pb	Zn	Cd	Ni	Sb	Cr
Minimum	111.7	331.4	4726.3	39.6	57.9	150.5	171.1
Maximum	507.6	886.2	11,683.9	78.2	449.4	473.2	744.0
Mean	245.8	538.4	8351.0	57.6	131.7	314.5	346.6
S.D	243.0	611.3	3505.3	20.5	216.0	123.2	352.1

**Table 4:** Comparison of selected metals concentrations (mg/kg) in PM_2.5_ fractions of road dust in some major cities of Asia.

City				Elements				Fraction Size
	Cu	Pb	Zn	Cd	Ni	Sb	Cr	
Karachi (This study)	332.9	426.6	4254.4	62.3	389.7	70.4	148.1	2.5 µm
Shikarpur (This study)	245.8	538.4	8351.0	57.6	131.7	314.5	346.6	2.5 µm
Dongying [42]	238.7	59.8	415.9	2.0	76.6	-	122.5	2.5 µm
Beijing [53]	160	260	590	10	50	-	100	2.5 µm
Incheon [54]	830	510	4450	20	60	270	560	2.5 µm
Hong Kong [55]	695	1209	5923	141	42	122	265	2.5 µm

**Table 5:** Heavy metals related to exhaust and non-exhaust emissions of vehicles reported in the literature.

Metals	Source of Emission	Reference
Cu	Brake wear, brake pad	(Pant et al.) [60]
Pb	Fuel, motor oil combustion, brake wear, resuspension of road dust	(Lough et al.) [10]
Zn	Brake wear, tyre abrasion, lubricants	(Hwang et al.) [61]
Cd	Brake wear, engine wear, auto printing	(Gope et al.) [62]
Ni	Fuel combustion, tyre abrasion	(Dehghani et al.) [63]
Sb	Brake wear, tyre wear, motor bearings, tailpipe emissions	(Fujiwara et al.) [64]
Cr	Combustion of lubricants and fuel	(Ubaid et al.) [1]

**Table 6:** Enrichment factor of heavy metals in road dust of Karachi (K.S: Karachi school area; K.H: Karachi hospital area; K.R: Karachi residential area; K.O: Karachi offices area).

Sample ID	Cu	Pb	Zn	Cd	Ni	Sb	Cr
K.S 1	25.15	5.50	21.87	5.56	9.72	104	8.74
K.S 2	1.95	2.50	10.82	2.41	4.98	40.79	4.47
K.H 1	12.61	11.05	67.45	15.37	20.41	187.80	25.30
K.H 2	3.98	8.75	21.34	5.05	9.78	78.33	7.19
K.R 1	8.60	12.66	54.18	14.46	24.06	204.22	13.76
K.R 2	8.14	6.05	37.31	7.75	11.12	104.64	12.43
K.O 1	8.37	7.448	37.68	10.79	19.16	140.85	12.51
K.O 2	4.52	4.31	25.97	5.76	9.65	78.83	7.46

**Table 7:** Enrichment factor of heavy metals in road dust of Shikarpur (S.S: Shikarpur school area; S.H: Shikarpur hospital area; S.R: Shikarpur residential area; S.O: Shikarpur offices area).

Sample ID	Cu	Pb	Zn	Cd	Ni	Sb	Cr
S.S 1	3.07	5.89	38.41	5.34	2.18	697.11	57.67
S.S 2	5.66	9.33	60.42	8.09	2.36	1004.77	40.83
S.H 1	7.031	11.45	93.34	10.01	15.69	1183.13	25.70
S.H 2	13.98	8.56	53.16	6.62	2.02	583.05	13.26
S.R 1	5.417	9.76	83.15	7.37	3.04	795.67	15.11
S.R 2	5.76	7.45	73.38	8.39	3.73	758.26	21.79
S.O 1	8.57	15.76	94.96	10.54	4.76	891.97	21.17
S.O 2	4.65	8.37	46.15	5.75	3.00	376.33	19.40

**Table 8:** Geoaccumulation Index (*I*_geo_) of heavy metals in road dust of Karachi.

Statistical Values	Heavy Metals						
**Geoaccumulation Index (*I*_geo_)**	**Pb**	**Cu**	**Cd**	**Ni**	**Cr**	**Zn**	**Sb**
**Minimum**	0.73	0.38	0.68	1.73	1.57	2.85	5.34
**Maximum**	3.07	4.06	3.35	4.00	4.07	5.49	7.67
**Mean**	1.98	2.61	2.48	3.18	2.93	4.52	6.87

**Table 9:** Geoaccumulation Index (*I*_geo_) of heavy metals in road dust of Shikarpur.

Statistical Values	Heavy Metals						
**Geoaccumulation Index (*I*_geo_)**	**Pb**	**Cu**	**Cd**	**Ni**	**Cr**	**Zn**	**Sb**
**Minimum**	1.97	1.03	1.83	0.4.3	3.14	4.67	7.97
**Maximum**	3.39	3.22	2.81	3.38	5.26	5.98	9.62
**Mean**	2.67	2.17	2.37	1.61	4.16	5.49	9.03

**Table 10:** Detailed description of factors of carcinogenic and non-carcinogenic indices.

Factor	Parameter	Unit	Values for Children and Adults	Reference
**AT**	Average Time	Days	365 × ED	(USEPA, 1989) (Mohmand et al.) [74]
**BW (Child)**	Bodyweight	Kg	28.4	(Rehman et al.) [27]
**BW (Adult)**	Bodyweight	Kg	61.5	(Ghanavati et al. 2019) [75]
**ED (Child)**	Exposure duration	Years	6	(Rehman et al. 2020) [27] (USEPA, 2001)
**ED (Adult)**	Exposure duration	Years	24	(Ghanavati et al. 2019) [75]
**EF**	Exposure frequency	Days/year	230	(Chen et al. 2014) [76]
**R_Inh_ (Child)**	Dust inhalation rate	m^3/^ day	7.63	(Mohmand et al. 2015) [74]
**R_Inh_ (Adult)**	Dust inhalation rate	m^3/^ day	20	(Ghanavati et al. 2019) [75]
**PEF**	Particular emission rate	m^3/^ kg	1.36 × 10^9^	(USEPA, 2001) (Mohmand et al. 2015) [74]

## Supplementary Materials

The following are available online at https://www.mdpi.com/1660-4601/17/19/7124/s1, Table S1: Reference dose and slope factor values used for calculation of hazard indices, Table S2: Non-carcinogenic and carcinogenic risk indices of toxic heavy metals in PM2.5 fraction of road dust samples, Table S3: Calculated values of Hazard Index (HI) for non-carcinogenic and carcinogenic heavy metals in PM2.5 fraction of road dust samples, Table S4: Classifications of geoaccumulation index (Muller, 1969), Table S5: Classifications of enrichment factor index, Figure S1: Resuspension chamber with PM2.5 size selective cutter, Figure S2: Sampling scheme for the preparation of PM2.5 fractioned road dust.
