# Supplementary material for: Health Risk Assessment of Heavy Metals Accumulated on PM2.5 Fractioned Road Dust from Two Cities of Pakistan
Source: Int J Environ Res Public Health. 2020 Sep 29;17(19):7124. doi: 10.3390/ijerph17197124 (PMC7579555; doi:10.3390/ijerph17197124)
Supplement: Supplementary file 1 [file ijerph-17-07124-s001.pdf]

Supplementary data

Health risk assessment of heavy metals accumulated on $PM_{2.5}$ fractioned road dust from two cities of Pakistan.

Haseeb Tufail Moryani[a], Shuqiong Kong[a,*], Jiangkun Du[a], Jianguo Bao[a,**]

[a] School of Environmental Studies, China University of Geosciences, Wuhan 430074, P.R. China

Table S1. Reference dose and slope factor values used for calculation of hazard indices (Rehman et al. 2020)[1] [2]

	Cd	Cr	Cu	Ni	Pb	Zn	Sb
**RfD_{Inh}**	1.00E-03	2.86E-05	4.02E-02	2.06E-02	3.52E-03	3.00E-01	
**SF**	6.30E+00	4.20E+01	-	8.40E-01	8.50E-03	-	7.10E-04

Table S2. Non-carcinogenic and carcinogenic risk indices of toxic heavy metals in PM2.5 fraction of road dust samples.

		Karachi															
		**Children**	**Adults**	**Children**	**Adults**	**Children**	**Adults**	**Children**	**Adults**	**Children**	**Adults**	**Children**	**Adults**	**Children**	**Adults**	**Children**	**Adults**
		**K.S1**	**K.S1**	**K.S2**	**K.S2**	**K.H1**	**K.H1**	**K.H2**	**K.H2**	**K.R1**	**K.R1**	**K.R2**	**K.R2**	**K.O1**	**K.O1**	**K.O2**	**K.O2**
**Pb**	HQ_{Inh}	1.09E-07	4.54E-06	4.97E-05	2.06E-06	2.2E-05	9.13E-06	1.74E-05	7.23E-06	2.52E-05	1.05E-05	1.2E-05	5E-06	1.48E-05	6.15E-06	8.57E-06	3.56E-06
	CR	3.275E-10	1.36E-10	1.49E-10	6.17E-11	6.58E-10	2.73E-10	5.21E-10	2.16E-10	7.54E-10	3.13E-10	3.6E-10	1.5E-10	4.43E-10	1.84E-10	2.57E-10	1.06E-10
**Cu**	HQ_{Inh}	2.828E-06	1.17E-06	2.2E-07	9.12E-08	1.42E-06	5.89E-07	4.48E-07	1.86E-07	9.67E-07	4.01E-07	9.16E-07	3.8E-07	9.41E-07	3.91E-07	5.08E-07	2.11E-07
	CR	-	-	-	-	-	-	-	-	-	-	-	-	-	-	-	-
**Cd**	HQ_{Inh}	5.14E-06	2.13E-06	2.23E-06	9.27E-07	1.42E-05	5.9E-06	4.67E-06	1.94E-06	1.34E-05	5.54E-06	7.16E-06	2.97E-06	9.97E-06	4.14E-06	5.32E-06	2.21E-06
	CR	3.239E-08	1.34E-08	1.41E-08	5.84E-09	8.95E-08	3.71E-08	2.94E-08	1.22E-08	8.42E-08	3.49E-08	4.51E-08	1.87E-08	6.28E-08	2.61E-08	3.35E-08	1.39E-08
**Ni**	HQ_{Inh}	1.68E-06	6.98E-07	8.62E-07	3.58E-07	3.53E-06	1.47E-06	1.69E-06	7.03E-07	4.16E-06	1.73E-06	1.92E-06	7.99E-07	3.32E-06	1.38E-06	1.67E-06	6.93E-07
	CR	2.911E-08	1.21E-08	1.49E-08	6.19E-09	6.11E-08	2.54E-08	2.93E-08	1.22E-08	7.21E-08	2.99E-08	3.33E-08	1.38E-08	5.74E-08	2.38E-08	2.89E-08	1.2E-08
**Cr**	HQ_{Inh}	4.91E-04	2.04E-04	2.51E-04	1.04E-04	1.42E-03	5.90E-04	4.04E-04	1.68E-04	7.73E-04	3.21E-04	6.98E-04	2.90E-04	7.02E-04	2.92E-04	4.19E-04	1.74E-04
	CR	5.897E-07	2.45E-07	3.02E-07	1.25E-07	1.71E-06	7.08E-07	4.85E-07	2.01E-07	9.28E-07	3.85E-07	8.39E-07	3.48E-07	8.44E-07	3.5E-07	5.04E-07	2.09E-07
**Zn**	HQ_{Inh}	1.12E-06	4.63E-07	5.53E-07	2.29E-07	3.44E-06	1.43E-06	1.09E-06	4.52E-07	2.77E-06	1.15E-06	1.90E-06	7.9E-07	1.92E-06	7.98E-07	1.33E-06	5.5E-07
	CR	-	-	-	-	-	-	-	-	-	-	-	-	-	-	-	-
**Sb**	HQ_{Inh}	-	-	-	-	-	-	-	-	-	-	-	-	-	-	-	-
	CR	5.51E-12	2.28E-12	2.16E-12	8.97E-13	9.95E-12	4.13E-12	4.15E-12	1.72E-12	1.08E-11	4.49E-12	5.54E-12	2.30E-12	7.46E-12	3.09E-12	4.18E-12	1.73E-12
		**Shikarpur**															

		Children	Adults	Children	Adults	Children	Adults	Children	Adults	Children	Adults	Children	Adults	Children	Adults	Children	Adults
		**S.S1**	**S.S1**	**S.S2**	**S.S2**	**S.H1**	**S.H1**	**S.H2**	**S.H2**	**S.R1**	**S.R1**	**S.R2**	**S.R2**	**S.O1**	**S.O1**	**S.O2**	**S.O2**
**Pb**	HQ $_{Inh}$	3.134E-05	1.3E-05	1.67E-05	6.91E-06	1.17E-05	4.86E-06	1.86E-05	7.71E-06	1.94E-05	8.06E-06	1.48E-05	6.15E-06	2.28E-05	9.45E-06	1.7E-05	7.07E-06
	CR	9.377E-10	3.89E-10	4.98E-10	2.07E-10	3.51E-10	1.46E-10	5.56E-10	2.31E-10	5.81E-10	2.41E-10	4.43E-10	1.84E-10	6.82E-10	2.83E-10	5.1E-10	2.11E-10
**Cu**	HQ $_{Inh}$	9.647E-07	4E-07	5.23E-07	2.17E-07	3.46E-07	1.44E-07	6.37E-07	2.64E-07	6.09E-07	2.53E-07	6.48E-07	2.69E-07	7.91E-07	3.28E-07	1.57E-06	6.52E-07
	CR	-	-	-	-	-	-	-	-	-	-	-	-	-	-	-	-
**Cd**	HQ $_{Inh}$	9.741E-06	4.04E-06	5.32E-06	2.21E-06	4.93E-06	2.05E-06	7.47E-06	3.1E-06	6.81E-06	2.83E-06	7.76E-06	3.22E-06	9.25E-06	3.84E-06	6.12E-06	2.54E-06
	CR	6.137E-08	2.55E-08	3.35E-08	1.39E-08	3.11E-08	1.29E-08	4.71E-08	1.95E-08	4.29E-08	1.78E-08	4.89E-08	2.03E-08	5.83E-08	2.42E-08	3.86E-08	1.6E-08
**Ni**	HQ $_{Inh}$	8.236E-07	3.42E-07	5.19E-07	2.16E-07	3.78E-07	1.57E-07	4.09E-07	1.7E-07	5.27E-07	2.19E-07	6.47E-07	2.68E-07	2.72E-06	1.13E-06	3.5E-07	1.45E-07
	CR	1.425E-08	5.91E-09	8.99E-09	3.73E-09	6.54E-09	2.71E-09	7.08E-09	2.94E-09	9.12E-09	3.78E-09	1.12E-08	4.64E-09	4.7E-08	1.95E-08	6.06E-09	2.52E-09
**Cr**	HQ $_{Inh}$	1.19E-03	4.93E-04	1.09E-03	4.52E-04	3.24E-03	1.34E-03	2.29E-03	9.52E-04	8.48E-04	3.52E-04	1.22E-03	5.08E-04	1.44E-03	5.99E-04	7.45E-04	3.09E-04
	CR	1.428E-06	5.93E-07	1.31E-06	5.43E-07	3.89E-06	1.61E-06	2.75E-06	1.14E-06	1.02E-06	4.23E-07	1.47E-06	6.1E-07	1.73E-06	7.19E-07	8.95E-07	3.71E-07
**Zn**	HQ $_{Inh}$	4.848E-06	2.01E-06	2.36E-06	9.78E-07	1.96E-06	8.14E-07	3.08E-06	1.28E-06	4.24E-06	1.76E-06	3.75E-06	1.55E-06	4.77E-06	1.98E-06	2.71E-06	1.13E-06
	CR	-	-	-	-	-	-	-	-	-	-	-	-	-	-	-	-
**Sb**	HQ $_{Inh}$	-	-	-	-	-	-	-	-	-	-	-	-	-	-	-	-
	CR	3.15E-11	1.30E-11	1.33E-11	5.52E-12	2.46E-11	1.02E-11	3.55E-11	1.47E-11	2.81E-11	1.16E-11	2.68E-11	1.11E-11	4.18E-11	1.73E-11	2.06E-11	8.55E-12

Table S3. Calculated values of Hazard Index (HI) for non-carcinogenic and carcinogenic heavy metals in PM2.5 fraction of road dust samples.

**City**		**Elements**						
**Karachi**		**Pb**	**Cu**	**Cd**	**Ni**	**Cr**	**Zn**	**Sb**
	**HI(Children)**	1.16E-04	8.25E-06	6.21E-05	1.88E-05	5.16E-03	1.41E-05	-
	**CR(Children)**	3.47E-09	-	3.91E-07	3.26E-07	6.20E-06	-	4.98E-11
	**HI(Adults)**	4.81E-05	3.42E-06	2.58E-05	7.82E-06	2.14E-03	5.86E-06	-
	**CR(Adults)**	1.44E-09	-	1.62E-07	1.35E-07	2.57E-06	-	2.06E-11
**Shikarpur**		**Pb**	**Cu**	**Cd**	**Ni**	**Cr**	**Zn**	**Sb**
	**HI(Children)**	1.52E-04	6.09E-06	5.74E-05	6.37E-06	1.21E-02	2.77E-05	-
	**CR(Children)**	4.56E-09	-	3.62E-07	1.10E-07	1.45E-05	-	2.22E-10
	**HI(Adults)**	6.32E-05	2.53E-06	2.38E-05	2.64E-06	5.01E-03	1.15E-05	-
	**CR(Adults)**	1.89E-09	-	1.50E-07	4.57E-08	6.02E-06	-	9.22E-11

Table S4. Classifications of geoaccumulation index (Muller, 1969)[3]

**Value**	**Category**
$\mathbf{I_{geo} \leqslant 0}$	unpolluted
$\mathbf{0 < I_{geo} \leqslant 1}$	unpolluted to moderately polluted
$\mathbf{1 < I_{geo} \leqslant 2}$	moderately polluted
$\mathbf{2 < I_{geo} \leqslant 3}$	moderately to strongly polluted
$\mathbf{3 < I_{geo} \leqslant 4}$	strongly polluted
$\mathbf{4 < I_{geo} \leqslant 5}$	strongly to extremely polluted
$\mathbf{I_{geo} > 5}$	extremely polluted

Table S5. Classifications of enrichment factor index.

**Value**	**Category**
EF~2	minimal enrichment
EF 2 – 5	moderate enrichments
EF 5 – 20	significant enrichments
EF 20 – 40	Very highly enriched
EF 40	extremely enriched

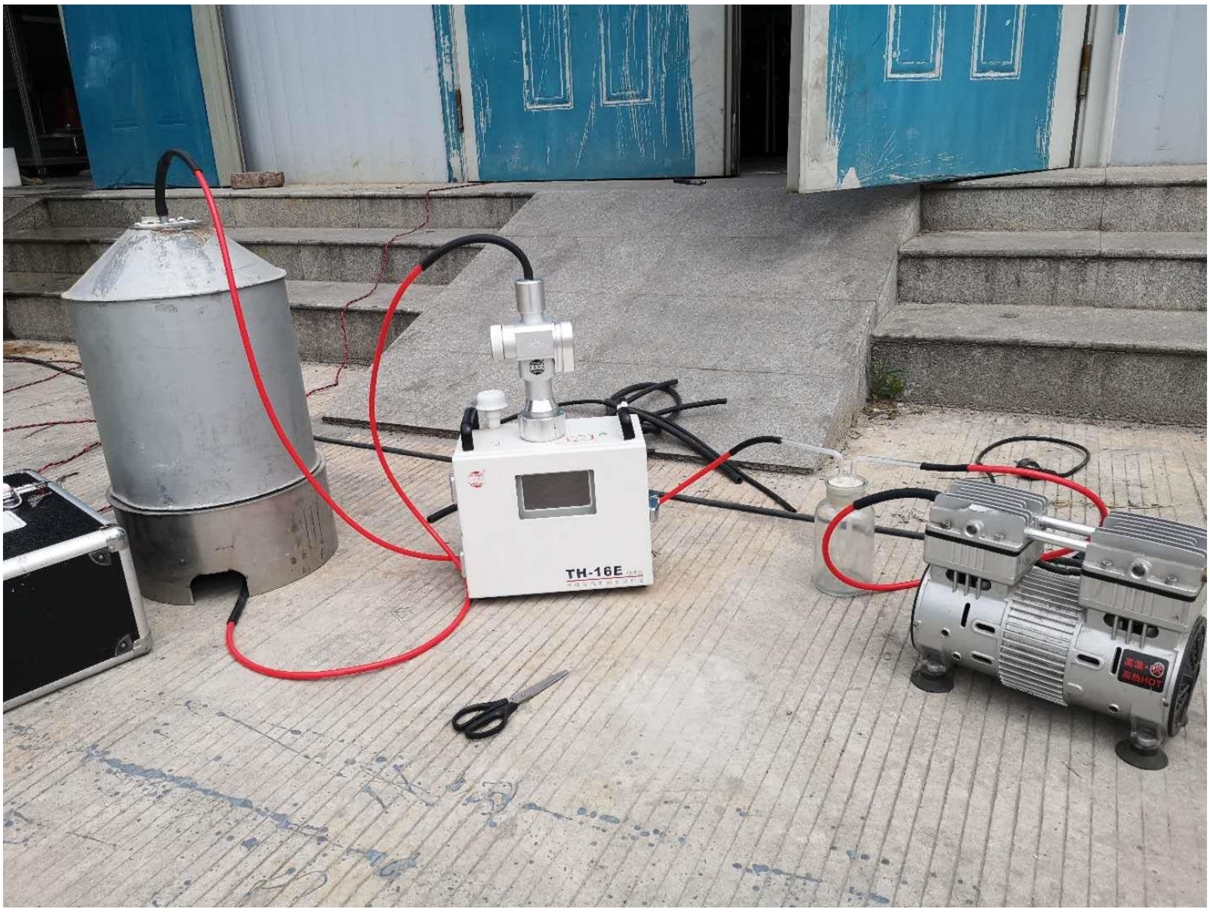

Figure S1. Resuspension chamber with $PM_{2.5}$ size selective cutter

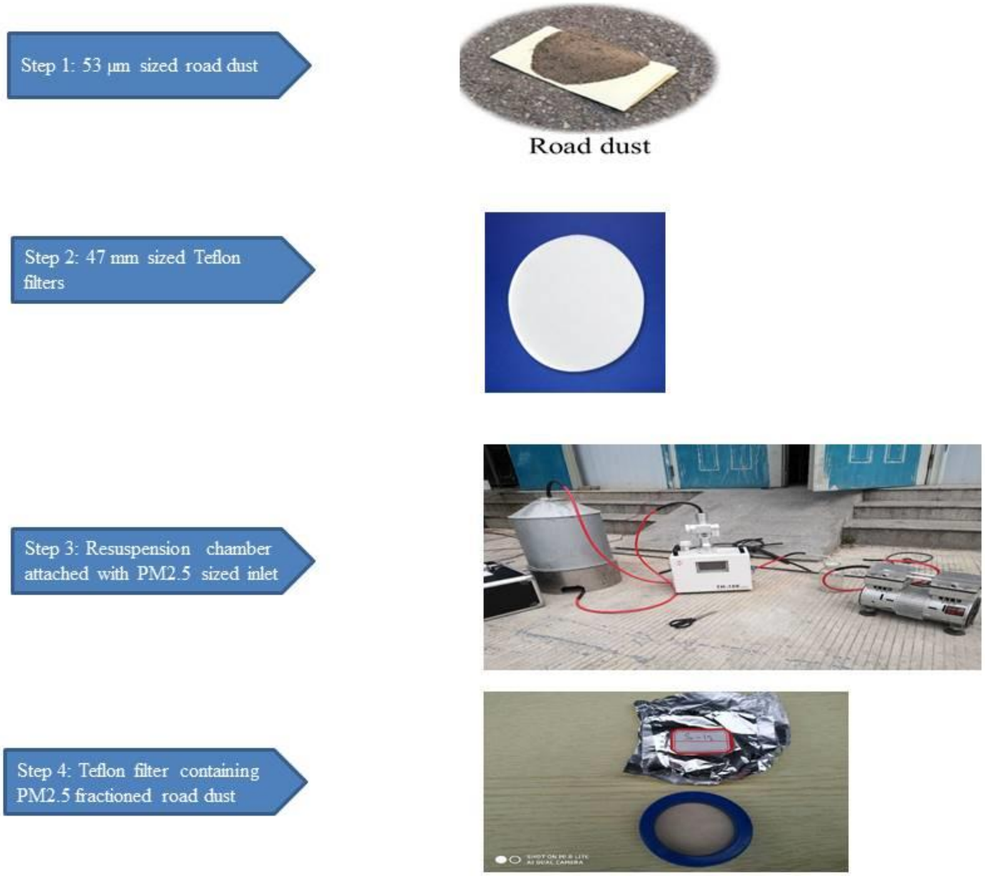

Figure S2. Sampling scheme for the preparation of $PM_{2.5}$ fractioned road dust.

References

1. Rehman, A.; Liu, G.; Yousaf, B.; Zia-ur-Rehman, M.; Ali, M.U.; Rashid, M.S.; Farooq, M.R.; Javed, Z. Characterizing pollution indices and children health risk assessment of potentially toxic metal(oid)s in school dust of Lahore, Pakistan. *Ecotoxicology and Environmental Safety* **2020**, *190*, 110059, doi:https://doi.org/10.1016/j.ecoenv.2019.110059.
2. Ramírez, O.; Sánchez de la Campa, A.M.; Sánchez-Rodas, D.; de la Rosa, J.D. Hazardous trace elements in thoracic fraction of airborne particulate matter: Assessment of temporal variations, sources, and health risks in a megacity. *Science of The Total Environment* **2020**, *710*, 136344, doi:https://doi.org/10.1016/j.scitotenv.2019.136344.
3. Muller, G. Index of Geoaccumulation in Sediments of the Rhine River. *Geo J.* **1969**, *2*, 109-118.
